# Long-term outcome upon treatment of calcified lesions of the lower limb using scoring angioplasty balloon (AngioSculpt™)

**DOI:** 10.1007/s00392-020-01610-3

**Published:** 2020-02-08

**Authors:** Mariya Kronlage, Carolin Werner, Matthias Dufner, Erwin Blessing, Oliver J. Müller, Britta Heilmeier, Hugo A. Katus, Christian Erbel

**Affiliations:** 1 Department of Cardiology, Angiology, Pneumology, University Hospital Heidelberg, Im Neuenheimer Feld 410, 69120 Heidelberg, Germany; 2 DZHK German Center for Cardiovascular Research, Partner Site, Heidelberg/Mannheim, Germany; 3 SRH Klinikum Karlsbad Langensteinbach, Guttmannstraße 1, 76307 Karlsbad, Germany; 4 Department of Internal Medicine III, University Hospital Schleswig-Holstein, Arnold-Heller-Straße. 3, 24105 Kiel, Germany; 5 Gefaesspraxis im Tal, Tal 13, 80331 Munich, Germany

**Keywords:** Percutaneous angioplasty, Scoring balloon, AngioSculpt™^®^, Limb ischemia, Calcified lesions, Drug-eluting balloon

## Abstract

**Aims:**

In peripheral artery disease (PAD), endovascular treatment success of heavily calcified lesions is often compromised by a number of vascular complications, such as recoils, dissections and need for target vessel re-interventions. The increasing use of scoring balloon techniques has raised the hope for better periprocedural outcomes; however, the knowledge regarding the actual benefits of the scoring balloon technique in comparison to standard therapy is still limited. Thus, the aim of the current study was to determine the safety and effectiveness of scoring balloon angioplasty in a real-life patients’ collective with PAD.

**Methods and Results:**

A total of 425 patients with moderate to severely calcified femoropopliteal lesions received interventional treatment between 2011 and 2018 at the single center; 230 received a treatment with a scoring balloon (AngioSculpt™), and 195 received a plain procedure without AngioSculpt™. Key questions of this analysis were: (1) whether AngioSculpt™ can be used as a safe and effective stand-alone treatment in heavily calcified lesions in a 24-month follow-up, as well as (2) whether target lesion preparation with scoring balloon bears additional benefits to standard treatment (PTA ± stent implantation). In terms of freedom from target lesion revascularization there were no significant differences between AngioSculpt™ and standard procedure (82.3% vs. 78.1%, *P* > 0.05). Vessel preparation with balloon angioplasty had no additional effects on survival and amputation rates in comparison to standard treatment without AngioSculpt™ (*P* > 0.05). The deployment of a scoring balloon did not reduce the subsequent need for additional stent implantations (32.6%, and 32.3%, *P* > 0.05).

**Conclusion:**

Lesion preparation with AngioSculpt™ scoring balloon represents a safe and effective tool in the treatment of complex femoropopliteal lesions. In this retrospective analysis, AngioSculpt™ scoring balloon angioplasty did not significantly improve vessel patency- both when used as an adjunctive in preparation for stenting and as stand-alone treatment. A prospective study is needed to further investigate the scoring balloon treatment options.

**Graphic abstract:**

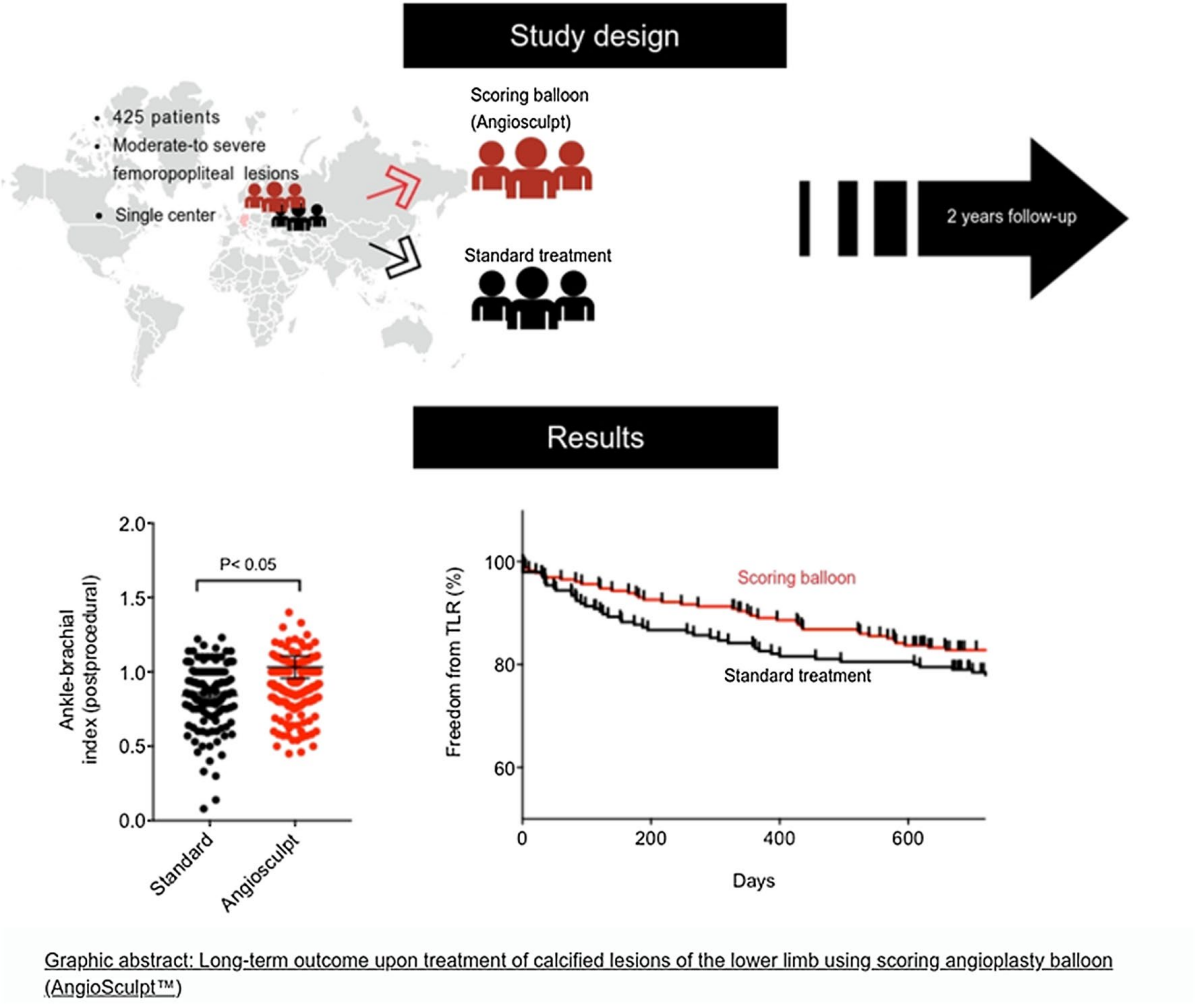

**Electronic supplementary material:**

The online version of this article (10.1007/s00392-020-01610-3) contains supplementary material, which is available to authorized users.

## Introduction

Despite the vast availability of different endovascular approaches in the therapy of peripheral artery disease (PAD) the treatment of chronic calcified lesions of the lower extremity remains a difficult task [1]. Although immediate revascularization success is given in the vast majority of cases, long-term patency rates are still modest [1–3]. Recoils upon standard percutaneous angioplasty (PTA) lead to the necessity of deploying additional devices, including atherectomy, as well as implantation of stents [4], with the consequence of longer procedure durations, material costs and radiation exposure, as well as a prolonged antithrombotic treatment regime and thus, bleeding risk [5]. Drug-eluting balloons (DEB) have also failed to markedly improve patency in such calcified lesions, probably due to insufficient diffusion of the antiproliferative medication through the barrier of the calcified vessel wall [6–9]. Hence, there is a valid notion that a careful indentation of the target lesion using scoring balloons with built-in helical nitinol elements a more atraumatic preparation and higher lumen gain of the target vessel could be achieved, thus minimizing the rate of periprocedural complications and additional procedures [10]. Although existing reports imply that AngioSculpt™ treatment is safe and associated with acceptable patency at 6 and 12 months [6], long-term evidence regarding efficacy of the device– as an adjunctive to standard procedures (DEB ± stent), as a stand-alone treatment option or as a tool for minimizing the need of additional stent implantation, are lacking.

## Methods

A total of 425 patients with predominantly calcified lesions of the lower extremity received interventional treatment and were continuously enrolled between 2011 and 2018 at the single center (University Hospital Heidelberg, Department of Cardiology, Angiology, Pulmology). Among those, 230 received a treatment with a scoring balloon (AngioSculpt™), whereas 195 received a plain procedure without AngioSculpt™. Target lesions were limited to the femoropopliteal region, in a total of 17 patients (6 in the non-Angiosculpt and 11 in the Angiosculpt group) the lesion extended towards the transition from the femoral to the external iliac artery. Follow-up examinations took place at the Heidelberg University Hospital and two associated outpatient angiology offices. They were performed immediately after, as well as in regular intervals up to 24 months after initial intervention; they included a clinical exam, ankle–brachial index measurement and a color duplex sonography, if indicated.

Primary efficacy outcome was defined as freedom from clinically driven target lesion revascularization (TLR) 24 months following the index procedure, as well as freedom from relevant re-occlusion (binary restenosis in the duplex sonography) of the target vessel (primary patency). In the duplex ultrasound, a peak systolic velocity ratio > 2.4 was used as a cutoff for detecting a > 50% stenosis; a peak systolic velocity ratio > 3.5 was the cutoff for a > 80% stenosis. Primary safety outcome was overall survival as well as freedom from target limb major amputation (amputation-free survival) at 24 months.

### Compliance with ethical standards

The study was approved by the local ethics committee at the University Hospital Heidelberg due to the retrospective design of the study as a part of the Heidelberg Registry for peripheral artery disease (S-331/2013); the registry was performed in accordance with the Declaration of Helsinki. Since only the responsible physicians had access to non-anonymized patient data, confidentiality of patient information was ensured.

### Interventional treatment methods

A total of 2500 IU heparin was administered after insertion of an introducer sheath in the femoral artery according to a standard protocol. Digital subtraction angiography (DSA) allowed the visual assessment of the grade of stenosis and severity of calcification upon which a decision for further procedure was taken by the interventionalist in charge. Lesion calcification was performed in conformity with the peripheral arterial research consortium (PARC) classification, with low calcification grade being focal isolated plaques and/or plaque conglomerate less than < 180° in the digital substraction angiography on one side of the vessel), moderate calcification grade being > 180° on both sides of the vessel, and severe classification being > 180° on both sides of the vessel (> 50% circumferential calcification) and more than the half of lesion length.

For standard balloon usage, a dilatation of the target lesion was performed with an adequate balloon size after passing the stenosis or occlusion. The inflation pressure was increased until full balloon inflation and held for 120 s. For scoring balloon usage, the stenosis was dilated by manometrically controlled inflation of the scoring balloon with the adequate size with a contrast agent and saline mixture by following the manufacturer´s instructions. Inflation pressure was increased by 2 atmospheres every 10-15 s until full balloon inflation was achieved and held for 60 s. After deflating and removing the standard or scoring balloon out of the vessel, a peripheral angiography was performed to control the effect of the dilatation. If there was a recoil of ≥ 30%, an additional dilatation with a standard balloon was initiated for about 120 s followed by a DSA. If there is still a recoil of ≥ 30% of the target lesion, a bail-out stent implantation was performed, followed by a post dilatation and a DSA. After completion of the intervention a DSA of the lower limb was performed to exclude a peripheral embolization.

Dual antiplatelet therapy (DAPT) with aspirin (100 mg/day) and clopidogrel (75 mg/day) was recommended for a minimum of 4 weeks following the procedure and aspirin (100 mg/day) alone thereafter, a prolonged DAPT regime was required in case a DEB/stent was used.

### Statistical analysis

Continuous variables were represented as mean ± standard error of the mean (SEM). Analysis of variance (ANOVA) method was used to compare means when variables were normally distributed. Chi-square tests were used to compare categorical data. Kaplan–Meier curves were drawn to present survival data and log-rank tests were used to assess differences in time to-event endpoints. *P* values of < 0.05 were considered statistically significant. Since no adjustment for multiplicity was performed, all *P* values need to be interpreted descriptively. In case of missing data, a complete-case analysis was performed. Data analysis was executed using GraphPad, Version 7.0 software.

## Results

### Study population

**Fig. 1:**
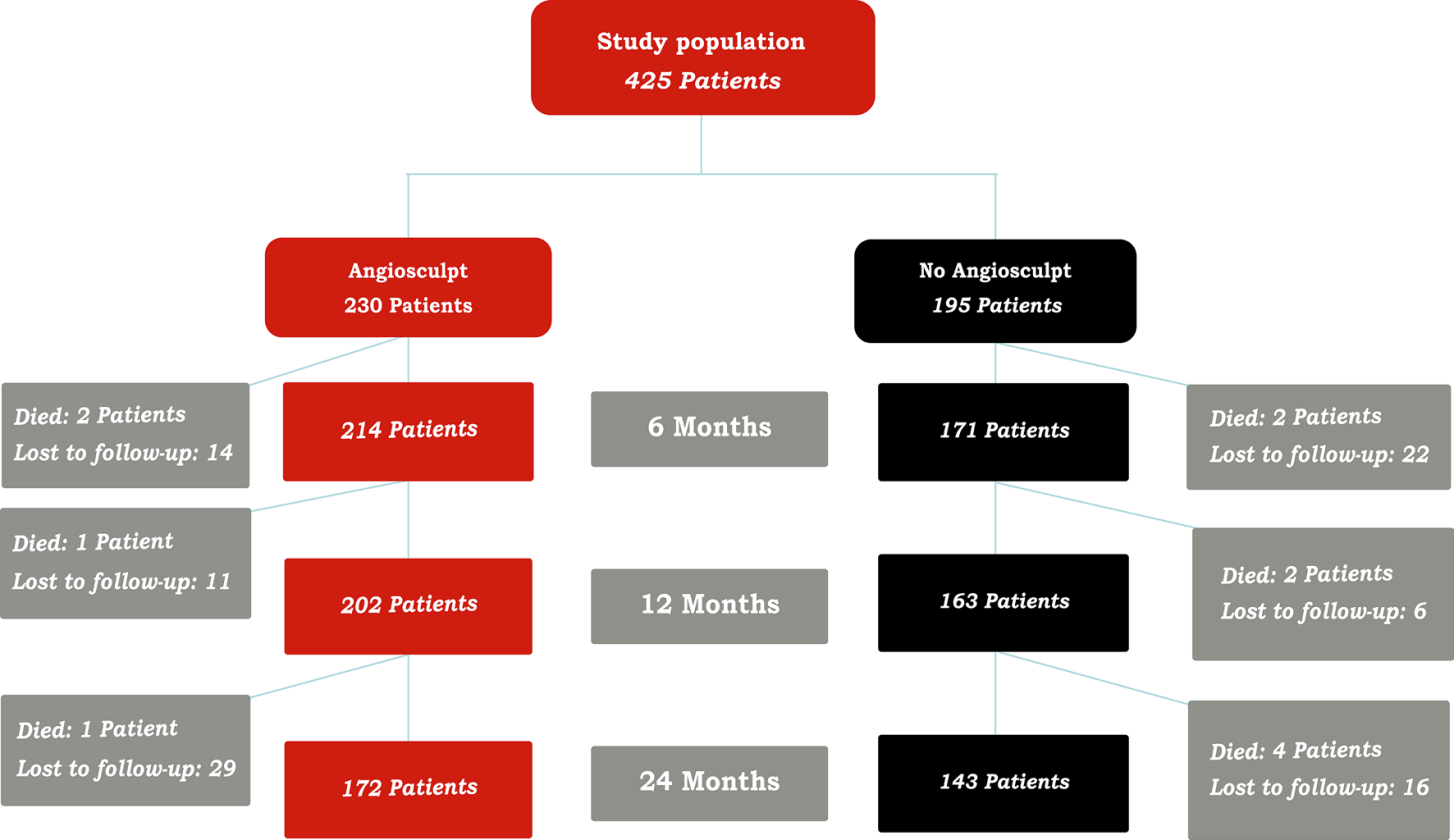
Flow chart of patients included in the study and lost to follow-up

A total of 425 patients have been included in the study in the period 2011–2018. The majority of patients in this real-life collective were male (169, 73.5% in the AngioSculpt™- and 118, 60.5% in the non-AngioSculpt™-treated group vs. 61, 26.5% females in the AngioSculpt™- and 77, 39.5% in the non-AngioSculpt™-treated group, *P* < 0.05), median age at time of procedure was 71 ± 10.4 years (Table 1). Patients presented with a variety of symptoms and a broad range of Rutherford stages; markedly, the duration of complaints was longer than 2 weeks in the majority of all cases (223/230, 96.7% in the AngioSculpt™ vs. 167/195, 85.6% in the non-AngioSculpt™ group, *P* < 0.05), Tables 2 and 3. A summary of the relevant co-morbidities and clinical data is presented in Table 4. Figure 1 represents a flow chart of the patients enrolled in the study and lost to follow-up.

**Table 1:** Demographic characteristics of the study population in both treatment groups

	Angiosculpt	No Angiosculpt
Gender (m)	169 (73.5%)	118 (60.5%) *
Age (years)	70.9 ± 10.1	71.1 ± 10.8^ns^
Weight (kg)	80.9 ± 16.3	75.9 ± 17.7 *
Height (cm)	171.7 ± 8.7	168.9 ± 12.3*

*ns* non-significant

*P < 0.05

**Table 2:** Rutherford stages at timepoint of admission as well as duration of symptoms prior to index procedure in both treatment groups

	Angiosculpt	No angiosculpt
Rutherford 2	42 (18.3%)	15 (7.7%) **
Rutherford 3	137 (59.6%)	86 (44.1%)**
Rutherford 4	8 (3.5%)	22 (11.3%)**
Rutherford 5	4 (1.7%)	4 (2.1%)^ns^
Rutherford 6	33 (14.3%)	58 (29.7%)**
Duration of complaints > 2 weeks	223 (97.0%)	167 (85.6%)**

Information was not retrievable for a total of 6 patients in the Angiosculpt group and 10 patients in the non-Angiosculpt group

*ns* non-significant

**P < 0.01

**Table 3:** Changes of Rutherford categories over time (baseline, as well as 6, 12 and 24 months follow-up)

Rutherford	Baseline	6 months	12 months	24 months
0 (asymptomatic)	*0*	*32 (18.7%)*	*30 (18.4%)*	*26 (18.2%)*
	**0**	**36 (16.8%)**	**34 (16.8%)**	**34 (19.8%)**
1 (mild)	*0*	*30 (17.5%)*	*47 (28.8%)*	*46 (32.2%)*
	**0**	59 (27.6%)*	**63 (31.2%)**	**65 (37.8%)**
2 (moderate)	*15 (7.7%)*	*49 (28.7%)*	*40 (24.5%)*	*16 (11.2%)*
	42 (18.3%)**	**74 (34.6%)**	**62 (30.7%)**	40 (23.2%)**
3 (severe)	*86 (44.1%)*	*16 (9.4%)*	*10 (6.1%)*	*25 (17.5%)*
	137 (59.6%)**	**20 (9.3%)**	**17 (8.4%)**	9 (5.2%)**
4 (rest pain)	*22 (11.3%)*	*2 (1.2%)*	*4 (2.5%)*	*4 (2.8%)*
	8 (3.5%)**	**4 (1.9%)**	**5 (2.5%)**	**6 (3.5%)**
5/6 (ulceration)	*62 (32.1%)*	*42 (24.6%)*	*32 (19.6%)*	*26 (18.2%)*
	37 (16%)**	21 (9.8%)**	21 (10.3%)*	18 (10.5%)*
Total				
non-Angiosculpt	*185*	*171*	*163*	*143*
Angiosculpt	**224**	**214**	**202**	**172**

Angiosculpt-treated cases are encoded in italic, non-Angiosculpt in bold. Information about the Rutherford category at baseline was not retrievable for a total of 6 patients in the Angiosculpt group and 10 patients in the non-Angiosculpt group

*P < 0.05, **P < 0.01

**Table 4:** Major clinical characteristics of the study population in both treatment groups

	Angiosculpt	No angiosculpt
Active smoking	64 (27.8%)	52 (26.7%)^ns^
Arterial hypertension	203 (88.3%)	176 (90.3%)^ns^
Diabetes mellitus type 2	96 (41.7%)	87 (44.6%)^ns^
Coronary artery disease	127 (55.2%)	75 (38.5%)**
COPD	12 (5.2%)	15 (7.7%)^ns^
Atrial fibrillation	30 (13%)	16 (8.2%)^ns^
GFR (ml/min)	64.4 ± 29.3	62.0 ± 28.6^ns^
Creatinine (mg/dl)	1.68 ± 1.9	1.62 ± 1.7 ^ns^
Hb (g/dl)	12.3 ± 2.5	12.2 ± 2.0 ^ns^

*ns* non-significant

**P < 0.01

### Lesion characteristics

The current report focuses primarily on femoropopliteal lesions of significant length (mean length in the Angiosculpt group 11.3 ± 5.9 cm, in the non-Angiosculpt group 10.9 ± 5.3 cm, ns). A thorough description of the lesion characteristics can be found in Table 5. In this real-life patients’ collective, AngioSculpt™-treated lesions had a significantly higher degree of calcification than non-AngioSculpt™-treated ones (140/195 vs. 91/230 with moderate to severe calcification *P* < 0.001, Table 5); consequently, the rate of (sub)total occlusions was higher than in the non-AngioSculpt™ group (139/195 vs. 88/230, *P* < 0.001).

**Table 5:** Lesion‘s characteristics

	Angiosculpt	No angiosculpt
Length (mean)	11.3 ± 5.9	10.9 ± 5.3 ^ns^
Calcification (moderate–severe)	140/230 (60.9%)	91/195 (39.5%)***
Lesion type		
De novo	131 (56.9%)	117 (60%)^ns^
Restenotic	73 (31.7%)	55 (28.2%)^ns^
In-stent restenotic	26 (11.3%)	23 (11.8%)^ns^
(Sub)total occlusion	139/230 (60.4%)	88/195 (45.1%)***
Intraprocedural vessel dissection	60/230 (26.0%)	39/195 (20%)^ns^
Run-off (post-procedural)		
Good	162 (70.4%)	123 (63.1%)^ns^
Compromised	53 (23%)	52 (26.7%)^ns^
Poor	15 (6.6%)	20 (10.3%)^ns^

*ns* non-significant

***P < 0.001

A summary of the additional devices (DEB, stents, atherectomy devices) used for target lesion revascularization has been listed in Suppl. Table 1.

### Clinical follow-up and patency

**Fig. 2:**
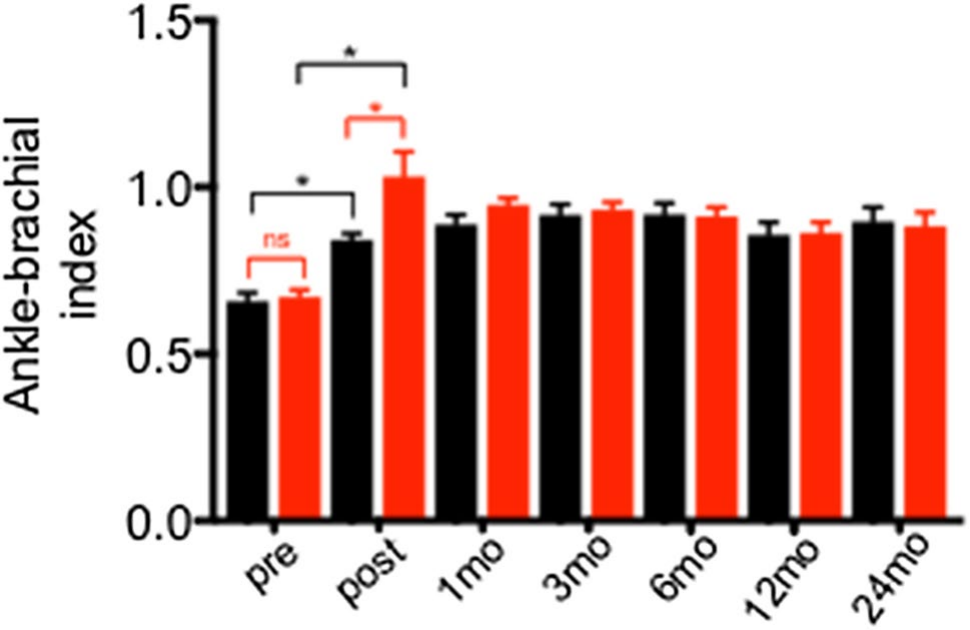
Values of ankle–brachial indices measured in both groups before (pre) and immediately after (post) procedure, as well as in regular intervals up to 24 months after index procedure. Black symbols indicate non-Angiosculpt-treated lesions, red symbols indicate Angiosculpt-treated ones. Intragroup statistical comparison for the values before and after intervention is indicated by black lines and mavericks, intergroup comparisons are highlighted in red. Abbreviations: mo: month, ns: non-significant, *P < 0.05

Upon revascularization, there was a substantial improvement of the ankle–brachial indices (ABI) in comparison to the values before intervention and AngioSculpt™-treated lesions showed a significantly better result than non-AngioSculpt™-treated ones immediately after treatment (*P* < 0.05), Fig. 2. During follow-up, both groups showed similar values; even 24 months upon index procedure, the ABIs remained stable, Fig. 2.

**Fig. 3:**
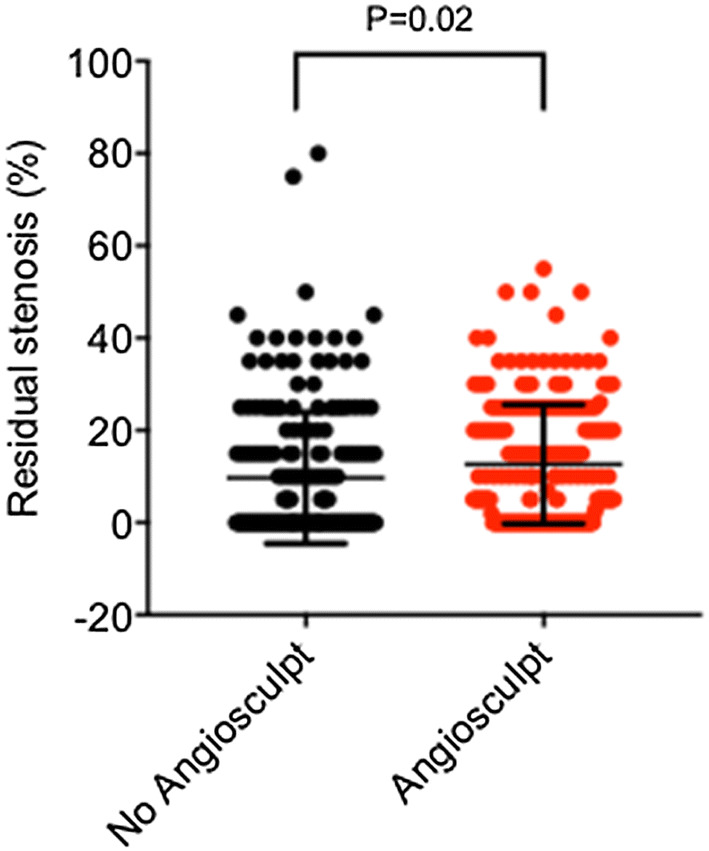
Dot blot of percentage of residual stenosis as determined from the digital subtraction angiographies. *P* value is indicated directly in the graph

Residual stenosis rate in the AngioSculpt™-treated lesions was 12.7% ± 12.9%, and in non-AngioSculpt™-treated lesions 9.7% ± 14.2%, *P* < 0.05, Fig. 3. Intraprocedural vessel dissection occurred in 39/195 vs. 60/230, *P* > 0.05) cases with no difference in the AngioSculpt™ vs. non-AngioSculpt™-treated lesions, Table 5; those were either minor or resolved via a consequent stent implantation. Local vascular complications at the puncture side were rare, with 1 case of AV-fistula, 2 aneurysma spurium, 1 vessel perforation that was treated via a covered stent implantation and 4 severe bleeding cases requiring blood transfusion. Major ultrasound characteristics over time can be found in Table 6.

**Table 6:** Stenosis rates of the target lesions over time (n baseline: 230 (AS)/195 (no AS); 6 months: 214 (AS), 171 (no AS); 12 months: 202 (AS), 163 (no AS), 24 months: 172 (AS), 143 (no AS)

Stenosis	Baseline		6 months		12 months		24 months	
	AS	No AS	AS	No AS	AS	No AS	AS	No AS
≤ 50%	–	–	186 (86.9%)	140 (81.9%)*	170 (84.2%)	132 (80.9%)^ns^	137 (79.7%)	106 (74.1%)^ns^
> 50%	17 (7.3%)	20 (10.2%)^ns^	14 (6.5%)	11 (6.4%)^ns^	16 (7.9%)	10 (6.1%)^ns^	15 (8.7%)	13 (9.1%)^ns^
> 80%	74 (32.2%)	61 (31.3%)^ns^	12 (5.6%)	17 (9.9%)^ns^	10 (5.0%)	15 (9.2%)^ns^	11 (6.4%)	10 (7.0%)^ns^
(Sub)total occlusion	139 (60.4%)	114 (58.5%)^ns^	2 (0.9%)	3 (1.8%)^ns^	6 (2.9%)	6 (3.9%)^ns^	9 (5.2%)	14 (9.7%)^ns^

*AS* angiosculpt, *no AS* no angiosculpt, *ns* not significant

*P < 0.05

**Fig. 4:**
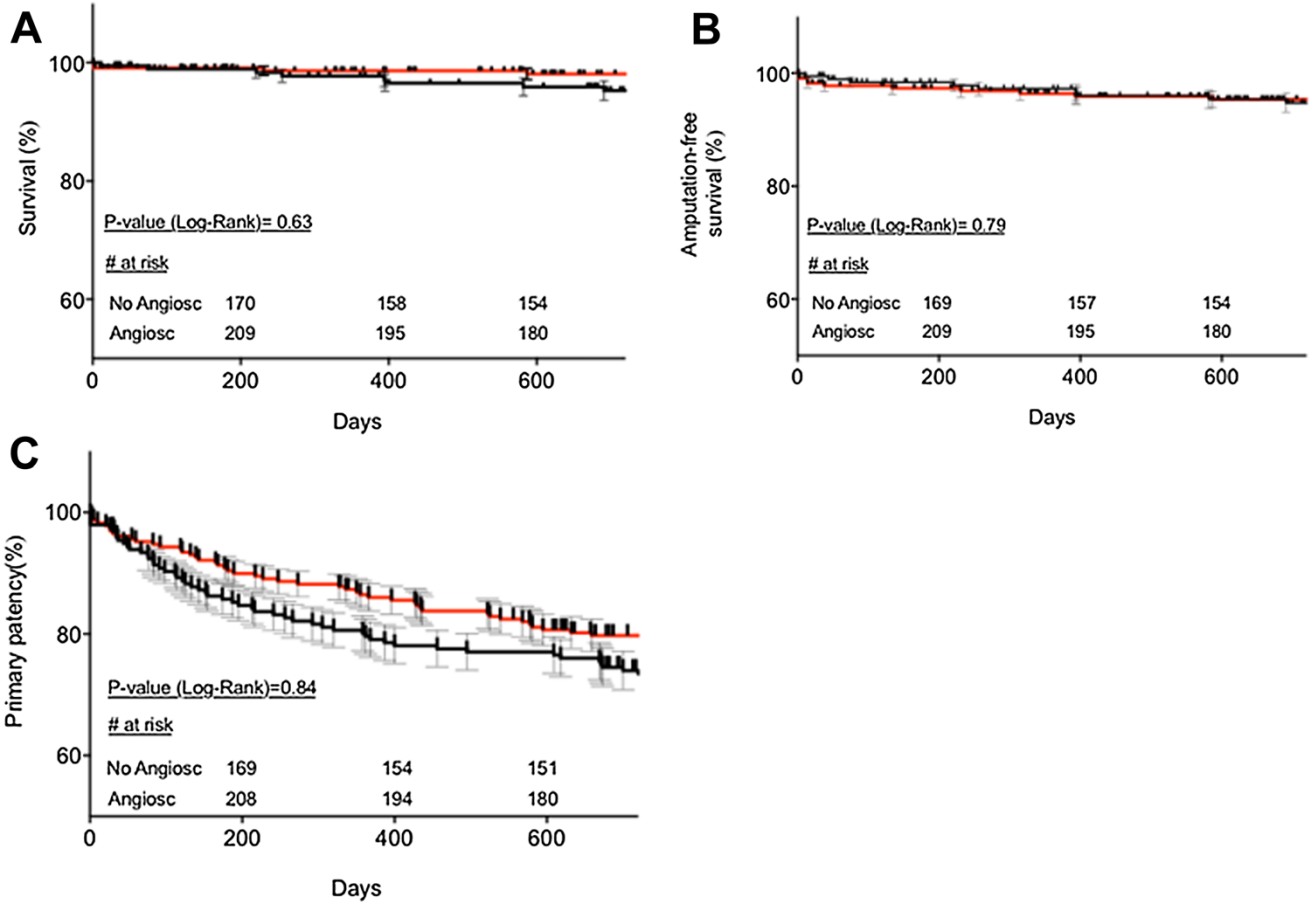
Kaplan–Meier analysis of overall survival (**a**) and amputation-free survival (**b**) and primary patency (**c**) in both groups. Black line indicates non-Angiosculpt-treated lesions, red line- Angiosculpt-treated ones. *P* values have been directly indicated in the graphs. Kaplan–Meier and standard error estimates (in %): **a** Timepoint 1 (day 200): Angiosculpt: 98.6 ± 0.9 vs non-Angiosculpt: 98.6 ± 0.8; Timepoint 2 (day 400):98.6 ± 0.9 vs. 96.5 ± 1.4; Timepoint 3 (day 600): 98.1 ± 0.9 vs. 95.9 ± 1.5. **b** Timepoint 1 (day 200): Angiosculpt: 96.8 ± 1.2 vs non-Angiosculpt:97.8 ± 1.1; Timepoint 2 (day 400):95.9 ± 1.3 vs 96 ± 1.5; Timepoint 3 (day 600): 95.4 ± 1.4 vs 95.4 ± 1.6. **c** Timepoint 1 (day 200): Angiosculpt: 89.5 ± 2.0 vs non-Angiosculpt: 83.7 ± 2.6; Timepoint 2 (day 400):85.6 ± 2.3 vs 78.1 ± 2.9; Timepoint 3 (day 600): 80.7 ± 2.6 vs 76.5 ± 3.0

Two years upon revascularization, overall and amputation-free survival rates in both groups remained > 95%, Fig. 4a, b. Primary patency appeared also with no significant difference between both groups in a 2-year follow-up (*P* > 0.05) (Fig 4c).

### Freedom from target lesion revascularization (TLR) and subsequent stent implantations

**Fig. 5:**
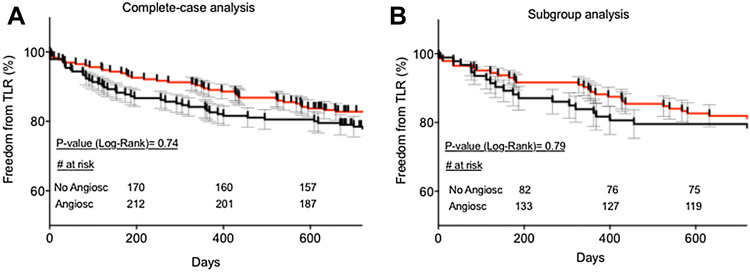
**a** Kaplan–Meier analysis of freedom from target lesion revascularization (TLR) in the full patients’ collective. **b** Kaplan–Meier analysis of freedom from TLR in the lesions with severe calcification only (subgroup analysis). Black line indicates non-Angiosculpt-treated lesions, red line—Angiosculpt-treated ones. *P* values have been directly indicated in the graphs. Kaplan–Meier and standard error estimates (in %): **a** Timepoint 1 (day 200): Angiosculpt: 92.1 ± 1.8 vs non-Angiosculpt: 86.7 ± 2.4; Timepoint 2 (day 400):88.6 ± 2.1 vs. 81.6 ± 2.8; Timepoint 3 (day 600): 83.7 ± 2.4 vs 80 ± 2.9, **b** Timepoint 1 (day 200): Angiosculpt: 91.7 ± 2.3 vs non-Angiosculpt: 87.1 ± 3.5; Timepoint 2 (day 400):87.5 ± 2.8 vs 80.6 ± 4.1; Timepoint 3 (day 600): 81.9 ± 3.2 vs 78.5 ± 4.3

Freedom from TLR was > 75% in both cases and there was no significant difference between AngioSculpt™ vs. non-AngioSculpt™- treated lesions (82.3% vs. 78.1%, *P* > 0.05), Fig. 5a, left panel (complete-case analysis). Since AngioSculpt™-treated lesions were characterized by a significantly higher calcification score, we further compared both treatments only in severely calcified lesions (> 50% circumferential calcification and more than the half of lesion length). In this subgroup, treatment with AngioSculpt™ was yet again not associated with a significant reduction in the rate of TLR (*P* > 0.05, Fig. 5b, right panel).

In addition, freedom from TLR remained unchanged, irrespective of a subsequent stent implantation, Suppl. Figure 1. Stent implantation rate was comparable between the groups (32.6% or 75/230 cases in the AngioSculpt™, and 32.3% or 63/195 cases in the non-Angiosculpt group, *P* > 0.05, Suppl. Figure 2).

## Discussion

Interventional treatment of heavily calcified lesions of the lower extremity remains a difficult task and patency rates of such lesions upon both standard and cutting balloon angioplasty remain moderate [7]. With the help of the built-in flexible nitinol wires of the scoring balloon that allow for a maximal focal concentration of the dilating force, there is the valid hope that a massively calcified vessel could be expanded in a less traumatic and more effective nature, possibly leading to better procedural results [11].

The use of scoring balloons in the coronary vessels has proven to have clear advantages, such as, e.g., to lead to a larger acute lumen gain and enhance stent expansion [11, 12]; however, scoring balloons have also been associated with a higher rate of major cardiovascular adverse events (MACE). In peripheral vessels, several reports have provided encouraging results regarding safety and practicability of AngioSculpt™ in different clinical settings [6, 10, 13, 14]; recently, it has been shown that primary patency after 12 months in PAD patients that underwent combination of DEB and AngioSculpt™ was comparable to patients that underwent scoring balloon angioplasty only [6].

To our knowledge, the current study provides the largest so far available analysis of patients with primarily calcified femoropopliteal lesions regarding both safety and efficacy of scoring balloon angioplasty in a long-term follow-up of 2 years upon index procedure. The study was designed to investigate two principle questions: (1) whether scoring balloon angioplasty is sufficient to improve patency and freedom from TLR in a long-term follow-up as a stand-alone treatment; and (2) are there and if yes- what are the additional benefits of AngioSculpt™-treatment on top of standard therapy (e.g., lumen gain, minimizing the rate of vessel dissections or the need of subsequent stent implantations due to recoil).

In our real-life collective, both overall survival and amputation-free survival rates reached > 95% in the two treatment groups. Immediately after procedure, AngioSculpt™-treated lesions presented with significant improvement in the ankle–brachial indices in comparison to non-AngioSculpt™-treated ones; however, the results in both treatment groups leveled up over the further follow-up of 24 months. Although results with AngioSculpt™ were slightly numerically superior to non-AngioSculpt™-treated cases in terms of both primary patency freedom from TLR, they failed to achieve a statistical significance.

Acknowledging the fact that AngioSculpt™-treated lesions had an overall higher calcification score, we performed a subgroup analysis and compared only lesions with a severe calcification. Although scoring alone is not sufficient to remove heavy calcium deposits, it is believed to facilitate breaking up relevant plaques and thus enhance further lesion preparation and subsequent procedural results, hence we wanted to investigate whether this effect is more prominent in severely calcified lesions. Even in this subgroup, scoring balloon angioplasty was not superior to standard therapy in terms of freedom from TLR. Our results also show that scoring balloon angioplasty did not have any significant advantageous effect in terms of residual stenoses, target vessel dissection rates and/or the need of subsequent stent implantations.

In summary, our data shows that AngioSculpt™ treatment is a safe interventional method that is associated with improvement of the immediate revascularization results, but bears no additional gain in the long-term follow-up in comparison to standard DEB therapy, irrespective of the fact whether additional stent implantation of the target lesion took place or not. In terms of efficacy endpoints, our results are concordant with previous reports regarding deployment of Angiosculpt™ in the endovascular therapy of peripheral lesions [6]. In the light of the preexisting evidence and our present data, scoring balloon angioplasty represents an additional option for atraumatic predilatation in highly calcified lesions of the lower extremity and can be considered, especially in lesions with high risk of dissection and recoil during intervention.

So far, there are no published randomized controlled trials comparing AngioSculpt™ to other strategies for lesion preparation for peripheral artery disease. Lessons from the domain of coronary artery disease (CAD) suggest that devices such as, e.g., rotational atherectomy might bear the advantages of compatibility with smaller sized catheters and facilitate the advancement of bigger-sized balloons towards the target lesion that can expand more effectively [15]; current investigations regarding intravasal lithotripsy in the femoropopliteal region have shown very promising results regarding the restoration of vessel compliance in severely calcified lesions and also bear the advantage of significantly minimizing the need for subsequent stent implantation [16]—an important aspect in which Angiosculpt™ treatment did not achieve superiority in the current study. Thus, future efforts should be directed at exploring the advantages of those devices over one another and at combining different devices to maximize procedural results and achieve better patency at larger patients’ cohorts. Emerging reports emphasize the advantages of such combination procedures [17, 18].

*Limitations* Although to our knowledge, the current study is the largest so far that investigates long-term follow-up upon scoring balloon angioplasty in peripheral vessels it underlies the limitations of a retrospective, single-center investigation. In addition, the choice of the corresponding interventional technique was done by the interventionist in charge and based on his or her personal assessment, thus it was not blinded or randomized. Notably, some variables were unevenly distributed, which might have an effect of the conclusions of the study.

## Electronic supplementary material

Below is the link to the electronic supplementary material.

Supplementary material 1 (PPTX 129 kb)
