# Supplementary material for: Long-term outcome upon treatment of calcified lesions of the lower limb using scoring angioplasty balloon (AngioSculpt™)
Source: Clin Res Cardiol. 2020 Feb 8;109(9):1177–85. doi: 10.1007/s00392-020-01610-3 (PMC7450001; doi:10.1007/s00392-020-01610-3)
Supplement: Supplementary file 1 — Supplementary material 1 (PPTX 129 kb) [file 392_2020_1610_MOESM1_ESM.pptx]

## Slide 1
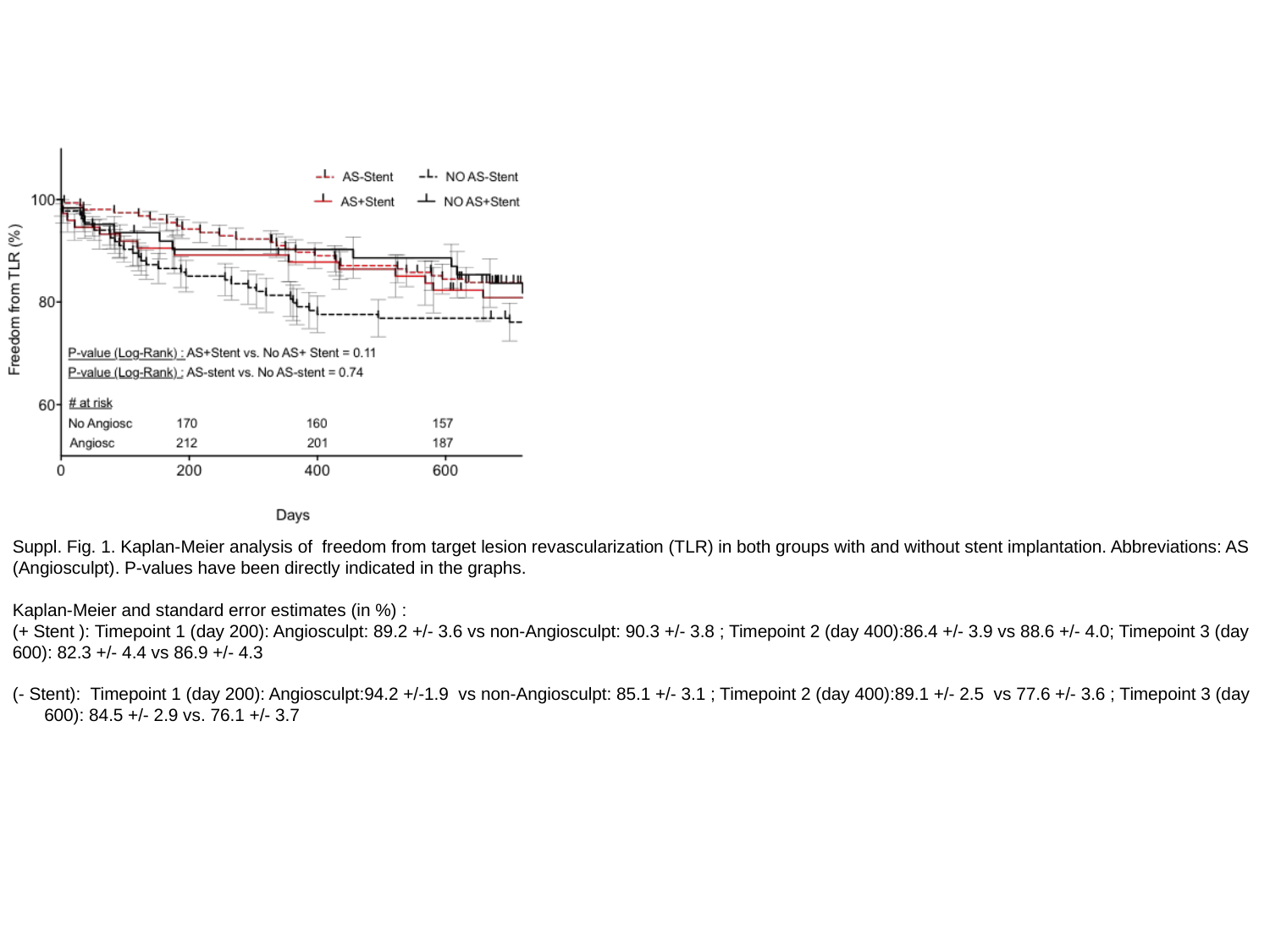

Suppl. Fig. 1. Kaplan-Meier analysis of freedom from target lesion revascularization (TLR) in both groups with and without stent implantation. Abbreviations: AS (Angiosculpt). P-values have been directly indicated in the graphs.
Kaplan-Meier and standard error estimates (in %) :
(+ Stent ): Timepoint 1 (day 200): Angiosculpt: 89.2 +/- 3.6 vs non-Angiosculpt: 90.3 +/- 3.8 ; Timepoint 2 (day 400):86.4 +/- 3.9 vs 88.6 +/- 4.0; Timepoint 3 (day 600): 82.3 +/- 4.4 vs 86.9 +/- 4.3
(- Stent): Timepoint 1 (day 200): Angiosculpt:94.2 +/-1.9 vs non-Angiosculpt: 85.1 +/- 3.1 ; Timepoint 2 (day 400):89.1 +/- 2.5 vs 77.6 +/- 3.6 ; Timepoint 3 (day 600): 84.5 +/- 2.9 vs. 76.1 +/- 3.7

## Slide 2
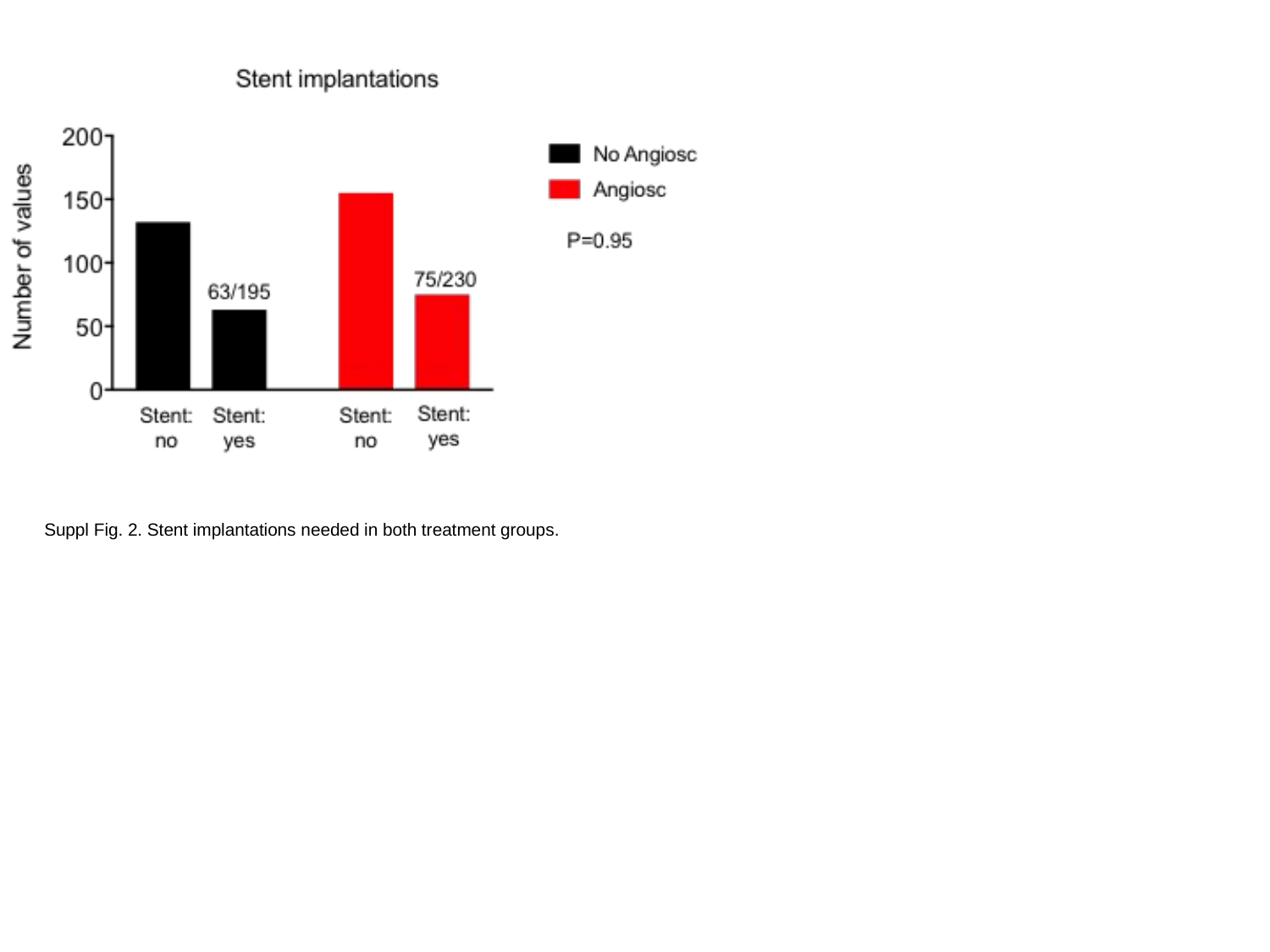

Suppl Fig. 2. Stent implantations needed in both treatment groups.

## Slide 3
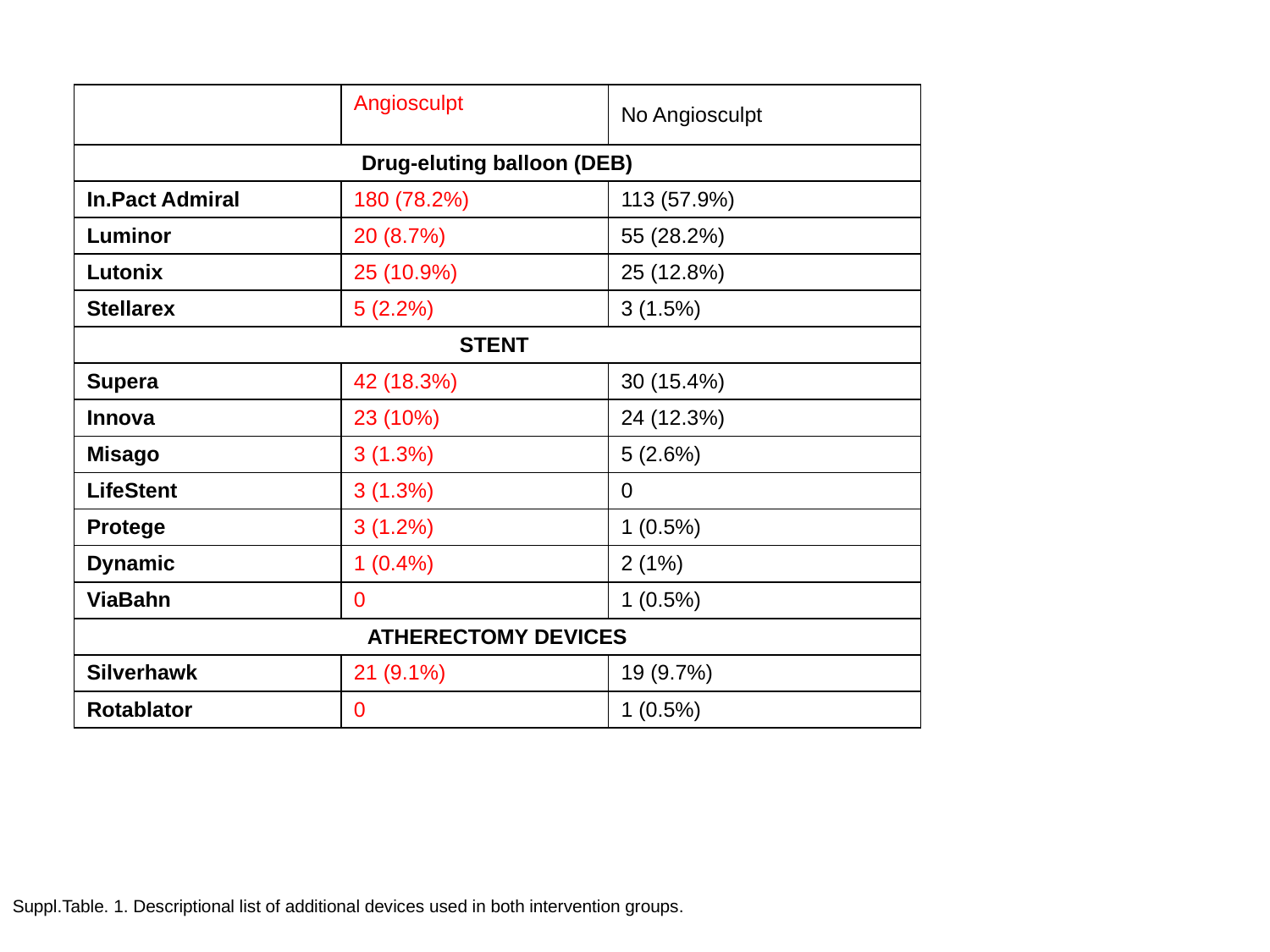

| | Angiosculpt | No Angiosculpt |
| --- | --- | --- |
| Drug-eluting balloon (DEB) | | |
| In.Pact Admiral | 180 (78.2%) | 113 (57.9%) |
| Luminor | 20 (8.7%) | 55 (28.2%) |
| Lutonix | 25 (10.9%) | 25 (12.8%) |
| Stellarex | 5 (2.2%) | 3 (1.5%) |
| STENT | | |
| Supera | 42 (18.3%) | 30 (15.4%) |
| Innova | 23 (10%) | 24 (12.3%) |
| Misago | 3 (1.3%) | 5 (2.6%) |
| LifeStent | 3 (1.3%) | 0 |
| Protege | 3 (1.2%) | 1 (0.5%) |
| Dynamic | 1 (0.4%) | 2 (1%) |
| ViaBahn | 0 | 1 (0.5%) |
| ATHERECTOMY DEVICES | | |
| Silverhawk | 21 (9.1%) | 19 (9.7%) |
| Rotablator | 0 | 1 (0.5%) |
Suppl.Table. 1. Descriptional list of additional devices used in both intervention groups.
